# Flexible Room-Temperature Ammonia Gas Sensors Based on PANI-MWCNTs/PDMS Film for Breathing Analysis and Food Safety

**DOI:** 10.3390/nano13071158

**Published:** 2023-03-24

**Authors:** Chonghui Zhu, Tingting Zhou, Hong Xia, Tong Zhang

**Affiliations:** State Key Laboratory of Integrated Optoelectronics, College of Electronic Science and Engineering, Jilin University, Changchun 130012, China

**Keywords:** flexible, gas sensor, PANI-MWCNTs/PDMS, NH_3_, breathing analysis

## Abstract

Gas sensors have played a critical role in healthcare, atmospheric environmental monitoring, military applications and so on. In particular, flexible sensing devices are of great interest, benefitting from flexibility and wearability. However, developing flexible gas sensors with a high sensitivity, great stability and workability is still challenging. In this work, multi-walled carbon nanotubes (MWCNTs) were grown on polydimethylsiloxane (PDMS) films, which were further modified with polyaniline (PANI) using a simple chemical oxidation synthesis. The superior flexibility of the PANI-MWCNTs/PDMS film enabled a stable initial resistance value, even under bending conditions. The flexible sensor showed excellent NH_3_ sensing performances, including a high response (11.8 ± 0.2 for 40 ppm of NH_3_) and a low limit of detection (10 ppb) at room temperature. Moreover, the effect of a humid environment on the NH_3_ sensing performances was investigated. The results show that the response of the sensor is enhanced under high humidity conditions because water molecules can promote the adsorption of NH_3_ on the PANI-MWCNTs/PDMS films. In addition, the PANI-MWCNTs/PDMS film sensor had the abilities of detecting NH_3_ in the simulated breath of patients with kidney disease and the freshness of shrimp. These above results reveal the potential application of the PANI-MWCNTs/PDMS sensor for monitoring NH_3_ in human breath and food.

## 1. Introduction

Ammonia (NH_3_), as an alkaline gas, can generate particulate ammonium sulfate and ammonium nitrate in the atmosphere, which cause PM2.5 pollution and serious lung diseases in humans [1,2,3]. According to the Occupational Safety and Health Administration (OSHA), the maximum concentration of NH_3_ to which a person is exposed should not exceed 30 ppm in 15 min; otherwise, it will seriously irritate human respiratory organs, skin and eyes. Moreover, NH_3_ in human exhaled gas is a sign of kidney disease. The concentration of NH_3_ in the exhaled gas of patients with kidney disease reaches 0.8–15 ppm, while the average NH_3_ concentration in the exhaled breath of healthy people is about 0.2–0.5 ppm [4,5]. This implies that there is a great potential for the use of NH_3_ sensors in the non-invasive diagnosis of kidney disease. Meanwhile, the production of fertilizers and the combustion of chemical industries also emit NH_3_ into the environment. Thus, the detection of NH_3_ is of great significance for both environmental protection and human health.

Among the different sensing materials, carbon nanotubes (CNTs) have received great attention in the field of gas sensing, especially for flexible gas sensors, because of their excellent electrical conductivity, chemical stability, high surface area and flexibility [6,7,8,9]. CNT-based gas sensors can generate charge transfer and induce a change in electrochemical signals when exposed to the target gas. For example, Lee et al. [10] fabricated flexible and transparent NH_3_ sensors based on functionalized single-walled carbon nanotube (SWCNT) film on PET substrates. The sensitivity of the sensor is 0.2% to 20 ppm NH_3_, and the response and recovery times are about 2 and 5 min, respectively. Penza et al. [11] used the RF plasma-enhanced chemical frontal vapor deposition technique to grow multi-walled carbon nanotubes (MWCNTs) on alumina substrates and loaded Pt particles on MWCNTs. The response value of the Pt-doped MWCNT-based sensor is 8.1% to 100 ppm NH_3_. Zhu et al. [12] modified SWCNTs with phosphomolybdic acid molecules, which were further connected to graphene-based electrodes by van der Waals forces. The response value is 11% to 5 ppm NH_3_, and the response time is 60 s. However, the sensing performance of CNTs is unsatisfactory because of the deficient charge transfer and weak interaction between CNTs and the target gas. Therefore, the development of CNT-based sensors with excellent performance and flexibility is still challenging.

Combining CNT materials with conductive polymers (polyaniline, polypyrrole, polythiophene, etc.) is a feasible method to construct gas sensors with a high sensitivity and selectivity [13,14,15,16,17,18,19]. On the one hand, the surface of the conducting polymer contains a mass of polar groups, which can enhance the interaction with carbon materials. This can improve electron transport and introduce new structure properties that are superior to those of pure-phase materials [20,21]. On the other hand, conductive polymers are suitable for wearable sensors because of their ease of processing, low cost and increased mechanical properties when compounded with carbon materials [22]. PANI, as a kind of conductive polymer, can selectively react with NH_3_ due to its unique electrical properties and doping/un-doping characteristics. Therefore, combining PANI and MWCNTs with optimal component ratios and structural design plays a key role in achieving flexible NH_3_ gas sensors with a high sensing sensitivity and stability. Wu et al. [23] prepared a porous polypropylene/CNT/PANI sensor to detect NH_3_. The response value of the sensor to 70 ppm NH_3_ is 452%, the response time is 93 s, and the detection limit is 500 ppb. Xue et al. [24] synthesized PANI nanoparticle-coated CNTs and PANI nanofibers and deposited them on polyethylene terephthalate (PET) substrates. The sensor can detect a range of NH_3_ concentrations from 200 ppb to 50 ppm. Wan et al. [25] prepared a flexible chemical gas sensor, which was assembled from PANI and CNT composites deposited on PET substrate. The response value of the flexible sensor is 30 to 100 ppm NH_3_, and the detection limit is 1 ppm. Although the reported PANI/CNT-based sensors have a high sensitivity, the detection limits do not meet the requirements of application in exhaled breath.

Herein, hydroxyl/carboxyl functionalized MWCNT nanomaterials were firstly deposited on a polydimethylsiloxane (PDMS) substrate. In addition, PANI was further loaded on the MWCNTs using a chemical oxidative polymerization method to construct a flexible PANI-MWCNTs/PDMS film. The surface of the acid-treated MWCNTs had oxygen-containing groups, which generated hydrogen bonds with the amino groups of the PANI chain to enhance the π-π conjugation interaction between them. The flexible PANI-MWCNTs/PDMS film could be directly assembled into gas sensors and exhibited excellent sensing performance to NH_3_ at room temperature. Furthermore, the sensors possessed great stability after bending or stretching cycles. The flexible sensor was also used in the detection of NH_3_ in breathing gas and food safety, and it demonstrated a superior sensing performance.

## 2. Experimental Section

### 2.1. Materials

Aniline (≥99.0%), multi-walled carbon nanotubes (MWCNTs, ≥95.0%, 10–50 μm) and ammonium persulfate (APS) were purchased from Aladding Reagent Co., Ltd. (Shanghai, China). Concentrated sulfuric acid (H_2_SO_4_) was purchased from Tianjing Komiou Chemical Regent Co., Ltd. Polydimethylsiloxane (PDMS) films were purchased from Alibaba Group, and their thickness was 0.1 mm.

### 2.2. Synthesis of PANI-MWCNTs/PDMS, MWCNTs/PDMS and PANI/PDMS Films

The synthesis of PANI-MWCNTs/PDMS films: APS was dispersed in 20 mL 1 mol·L^−1^ H_2_SO_4_. Next, 10 mg MWCNTs and PDMS film were added to the above solution. Then, the solution was stirred for 6 h at 60 °C. The MWCNT solution was cooled to room temperature. The purpose of this step is to modify MWCNTs to introduce surface functional groups, such as carboxyl (-COOH) or hydroxyl (-OH) groups. Then, 200 μL aniline was added to the MWCNT solution and stirred for 30 min. After that, the above solution was transferred to a 50 mL stainless steel reactor with Teflon lining at 100 °C for 4 h. Then, the PANI-MWCNT/PDMS films and powder were washed 3–5 times with ethanol and deionized water, respectively. The films fabricated by adding 0.1, 0.2 and 0.3 g APS to the reaction were named as PM-1/PDMS, PM-2/PDMS and PM-3/PDMS films, respectively.

The synthesis of MWCNTs/PDMS films: 0.2 g APS, 10 mg MWCNTs and PDMS were added to 20 mL H_2_SO_4_ (1 mol·L^−1^). The solution was stirred for 6 h at 60 °C to prepare MWCNTs/PDMS films.

The synthesis of PANI/PDMS films: APS was dispersed in 20 mL 1 mol·L^−1^ H_2_SO_4_. Then, 200 μL aniline was added to the solution and stirred for 30 min. The above solution was transferred to a 50 mL stainless steel reactor with Teflon lining at 100 °C for 4 h.

### 2.3. Characterization

The morphology of the materials was recorded using a field-emission scanning electron microscope (FESEM), model JSM-7500F (JEOL, Tokyo, Japan). The ultraviolet–visible (UV-vis) spectrum was recorded on a UV-3600 (Shimadzu, Tokyo, Japan), and the Fourier infrared (FT-IR) spectrum was measured using a Perkin–Elmer spectrometer. The Raman spectrum of the materials was measured using a LabRAM HR800 (Horiba Jobin Yvon, Palaiseau, France).

### 2.4. Fabrication of Gas Sensors

The MWCNTs/PDMS and PANI/PDMS PANI-MWCNT/PDMS films were cropped to prepare 1 × 1.5 cm^2^ thin films. Both ends of the films were clamped with two clips and connected to test lines to measure the sensing performance. The response of the sensors was defined as S = R_g_/R_a_. R_g_ and R_a_ are the resistance in the target gas and air, respectively. The exposure time and recovery time were defined as 100 s and R_g_-90%(R_g_-R_a_), respectively. Breath samples were obtained from six healthy volunteers, aged 24–30 years, and the experiment was performed with their consent. The operating temperature of the sensors was room temperature (23 ± 2 °C). The gas sensing performances of the sensors were investigated using a CGS-8 test system (Beijing, China). The relative humidity (RH) and gas concentration were controlled using a DGL-III gas and liquid distribution system (Elite Technology, Suzhou, China). Air and ammonia gas were used to condition the test gas concentration environment. The relative humidity was controlled via water evaporation, and the RH values were regulated to 10%, 20%, 40%, 60% and 80% via the high-precision control of the flow and temperature of the gas distribution system. A schematic of the sensor test system is shown in Supporting Information (Figure S1).

## 3. Results and Discussion

### 3.1. Morphology and Structure

**Figure 1:**
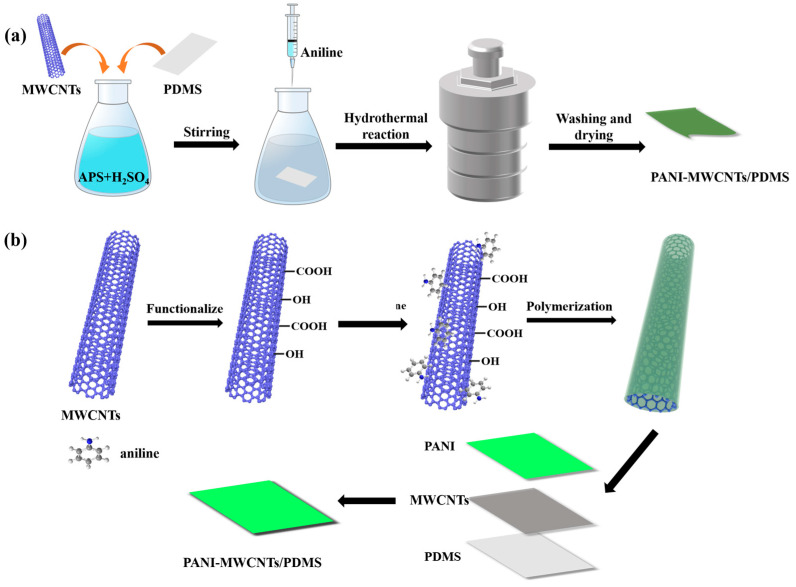
Schematic diagram of the preparation (**a**) and microstructure (**b**) of PANI-MWCNTs/PDMS films.

**Figure 2:**
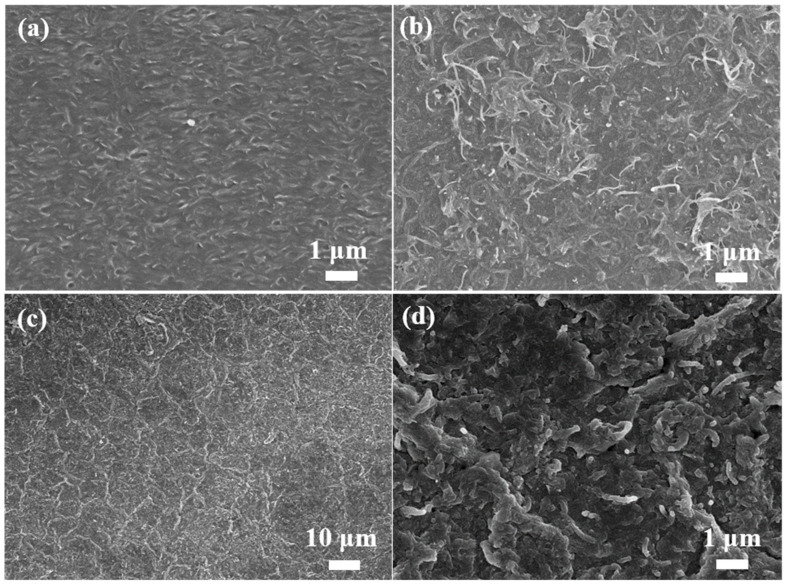
SEM images of the PDMS (**a**); MWCNTs-PDMS (**b**); PM2 films (**c**,**d**).

Figure 1 illustrates a schematic diagram of the preparation of the PANI-MWCNTs/PDMS films. In an acidic environment, the MWCNTs were modified by forming –COOH and –OH groups on the surface, and they were grown on PDMS films. It was observed that the color of the PDMS film changed from colorless to gray, which proved that the MWCNTs were successfully assembled onto the film (Figure S2a). Subsequently, aniline was added to prepare PANI and further deposited on the MWCNTs/PDMS films through electrostatic adsorption to form PANI-MWCNTs/PDMS.As shown in Figure S2b, the color of the film changed from gray to green. The SEM pictures show the microstructure of the PANI and MWCNTs grown on the PDMS films. The surface of the PDMS film was folded. When the film was loaded with MWCNTs, the MWCNTs were more uniformly dispersed on the film to form a network (Figure 2a,b). Moreover, the diameter of the MWCNTs was about 60 nm. The morphology of the PANI-MWCNTs/PDMS film was further observed. The results show that the surface of the PANI-MWCNTs/PDMS film has more wrinkles (Figure 2c). The surface of the PM1 film had more nanoparticles, with a diameter of 300–600 nm (Figure S3a,b). With the increase in the PANI content, the roughness of the film surface became larger, and the PANI materials gradually covered the MWCNTs. The nanoparticles gradually changed to nanorods. The surface of the PM2 film consisted of nanoparticles and nanorods with diameters of approximately 100 nm and 200 nm, respectively (Figure 2d). Moreover, the number of nanorods on the surface of the PM3 film increased, and the diameter increased to about 500 nm (Figure S3c,d).

**Figure 3:**
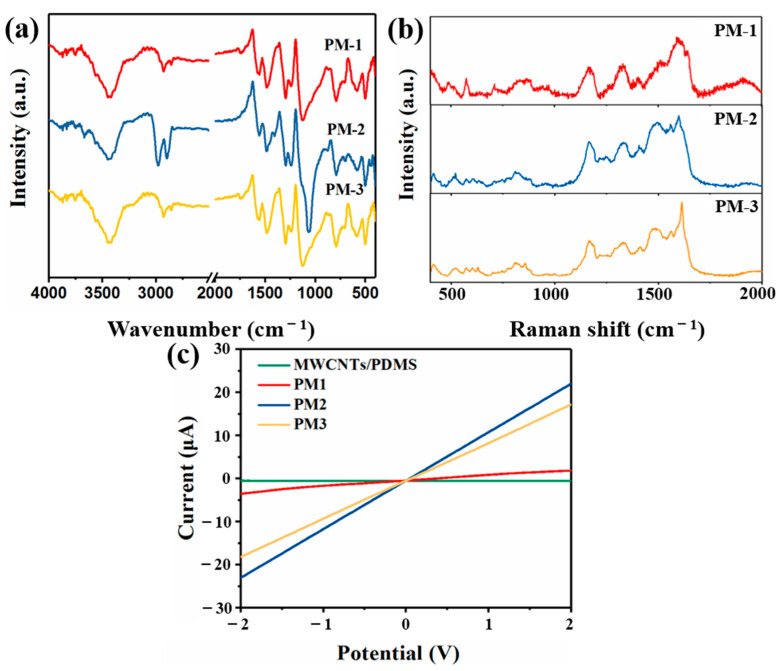
FTIR (**a**), Raman (**b**) and I-V curve (**c**) spectrograms of PANI-MWCNTs/PDMS films.

The FT-IR patterns of the PANI/MWCNTs-PDMS films are shown in Figure 3a. The adsorption band at 3442 cm^−1^ is designated as PANI N-H stretching vibrations. The peaks at 1558 and 1484 cm^−1^ are attributed to the C=C stretching of the quinoid rings and the C-C stretching of the benzenoid rings of PANI, respectively. The materials exhibit characteristic peaks at 1295, 1239, 1138 and 790 cm^−1^, which correspond to the absorption of aromatic amine, C-NH^+^ stretching, the in-plane C-H bending vibration of the quinone ring and the C-H bending vibration of 1,4-disubstituted benzene. This indicates that PANI formed in the acid-doped state [26,27,28]. The weak absorption peaks at 1752, 1659 and 705 cm^−1^ correspond to the symmetric stretching vibration of C=O, the -C=O- vibration and the deformation vibration of the acyl ring. These energy bands correspond to the CO-N-OC group, demonstrating that MWCNTs-COOH attached to PANI through covalent bonds [29,30,31]. The Raman spectra provide additional evidence for the growth of PANI along the MWCNTs. The MWCNTs/PDMS film exhibits two characteristic bands at 1588 and 1342 cm^2^, which are attributed to the high-frequency modes of the phonon (G mode) and the disorder-induced peak (D mode) (Figure S4). Moreover, the peaks at 490, 708, 2905 and 2965 cm^−1^ are the characteristic peaks of PDMS films. With the addition of the content of PANI, the peaks of the MWCNTs are weakened, and the peaks of PANI are displayed. The peaks at 1598, 1496, 1321 and 1160 are attributed to the C-C bond in benzene, N-H deformation, C-N^+^ bond stretching and the C-H bending of quinoid, respectively (Figure 3b) [27,32,33]. The synthesized films were cut to 1 × 1.5 cm^2^, and the I-V curves were tested, as shown in Figure 3c. The composite films exhibit an ohmic behavior in the [–2, +2] V range. Through a corresponding fitting with a power law of I = AU^n, the n values of the films are 0.0012, 1.38, 1.06 and 1.09. The result shows that the n value of the PM2 films is closer to 1. The MWCNTs/PDMS films have poor electrical conductivity, and PANI is a conducting polymer that can impart high electrical conductivity to the material. As the PANI content increases, the electrical conductivity of the films also increases. Furthermore, the PM2 film has better conductivity, which makes the electron mobility faster, and the delocalization effect of the material on polarons becomes stronger [34,35,36].

### 3.2. Gas Sensing Properties

**Figure 4:**
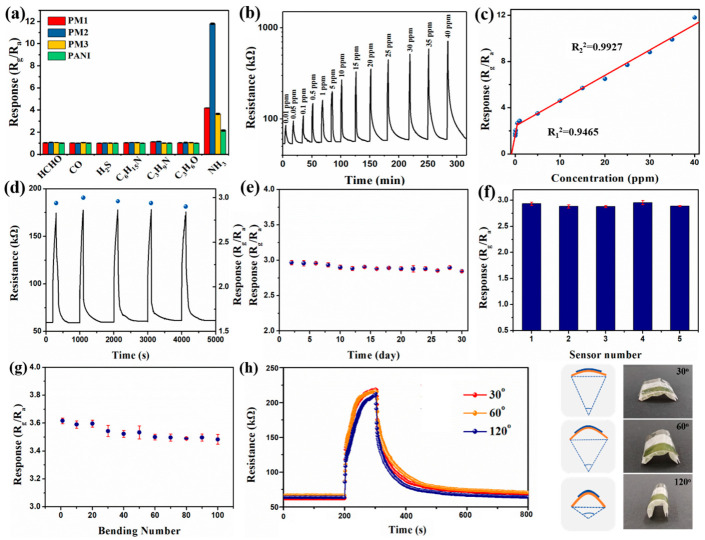
The response of sensors to 40 ppm of various gases (**a**); the response–recovery curve (**b**) and the response value (**c**) of PM2 sensor to 0.01–40 ppm NH_3_; the reproducibility (**d**), the long-term stability (**e**) and the sensor numbers (**f**) of PM2 sensor; the effect of bending number (**g**) and bending angle (**h**) on response to 5 ppm NH_3_.

In order to compare the sensing performances of the prepared materials, the sensors based on the PM1, PM2, PM3, pure PANI/PDMS and MWCNTs/PDMS films were exposed to 40 ppm of different gases at 23 ± 2 °C and 35 ± 2% RH (Figure 4a). Among them, the MWCNTs/PDMS film could not be monitored due to the high resistance values. The sensitivities of the used sensors to NH_3_ are much higher than those of formaldehyde (HCHO), carbon monoxide (CO), hydrogen sulfide (H_2_S), triethylamine (C_6_H_15_N), trimethylamine (C_3_H_8_N) and acetone (C_3_H_6_O). In addition, the PM2 film sensor exhibits the highest response to NH_3_. The response of the PM2 film sensor is 11.8 ± 0.2 to 40 ppm NH_3_ at room temperature. Figure 4b exhibits the dynamic response–recovery curve of the PM2 film sensor to 0.01–40 ppm NH_3_ at room temperature. The low detection limit of the sensor is 0.01 ppm, and the recovery time is 236 s (Figure S5). The exposure time is defined as 100 s due to the inability of the sensors to reach adsorption equilibrium. The PM2 sensor shows good linearity at 0.01–1 and 1–40 ppm NH_3_, and the linear correlation coefficients are 0.9465 and 0.9927, respectively (Figure 4c). In addition, most of the reported substrates for NH_3_ sensors are rigid materials, including interdigital electrodes, glass substrates, silicon (Si) substrates and ceramic tubes (Table 1). In contrast, the PANI-MWCNTS/PDMS sensors prepared in this work have good flexibility while having a good response (11.8 to 40 ppm) and a low detection limit (10 ppb).

The reproducibility of the PM2 sensor was studied via repeated exposure to 1 ppm NH_3_ at room temperature. The PM2 sensor exhibited good repeatability over five cycles with a relative standard deviation (RSD) of 1.23% (Figure 4d). Long-term stability is also one of the important parameters of sensors. As shown in Figure 4e, the response of the PM2 sensor to 1 ppm NH_3_ was continuously tested over 30 days. The value of RSD was 2.4%, which indicates that the sensor has superior long-term stability. In order to further investigate repeatability and stability, five PM2 film-based sensors were fabricated. The five devices had similar responses to 1 ppm NH_3_ (RSD = 2.5%). For flexible sensors, bending and stretching may affect their performance. Therefore, we investigated the effect of bending number and angle on the PM2 sensor. Figure 4g shows the effect of bending times on the PM2 sensor. As the number of bends increased, the response of the PM2 sensor to 5 ppm NH_3_ further decreased. After 100 bending times, the response of the sensor decreased by 3.9%. Then, the effect of the bending angle on the sensors was studied. When the bending angles were 30°, 60° and 120°, the response values of the PM2 sensor to 5 ppm NH_3_ were 3.5, 3.3 and 3.4, respectively (Figure 4h). All the above results show that the flexible sensor has good mechanical properties.

**Figure 5:**
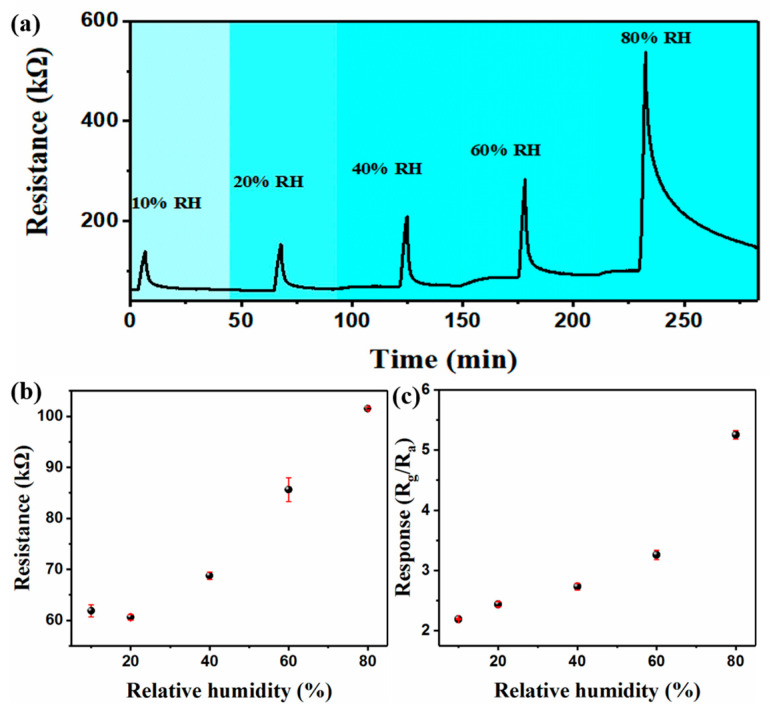
Sensing transients (**a**), initial resistance (**b**) and the response (**c**) of PM2 sensor to 1 ppm NH_3_ in humid environment.

The properties of NH_3_ gas sensors are greatly influenced by relative humidity in practical applications. Therefore, the NH_3_ sensing performance of the PM2 sensor was further investigated in the range of 10–80% RH (Figure 5a). The PM2 sensor was placed in different humidity environments, and when the resistance of the sensor reached a stable value, the sensor’s device was fed with 1 ppm NH_3_ to detect the change in the resistance. The results show that the resistance value of the sensor is almost unchanged, which is about 60 kΩ at 10–20% RH. When the humidity increases from 40% to 80% RH, the resistance of the PM2 sensor increases from 68 kΩ to 101 kΩ (Figure 5b). With the increase in RH, the response of the sensor increases. The response value of the sensor reaches 2.7 at 40% RH. When the RH is 80%, the response value of PM2 increases to 5.3, and the recovery time becomes longer. In order to exclude the influence of ambient humidity on the sensor, we placed the PM2 sensor in different relative humidity environments without NH_3_. The results show that the response of the PM2 sensor to different humidity environments does not exceed 1.5 (Figure S6). The above results indicate that the response of the PM2 sensor to NH_3_ increases in a high humidity environment.

### 3.3. Gas Sensing Mechanism

**Figure 6:**
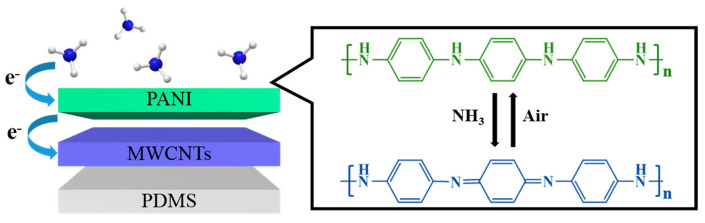
Gas sensing mechanism of PANI-MWCNTs/PDMS sensor.

The gas sensing mechanism of the PANI-MWCNTs/PDMS film sensors are mainly attributed to the unique doping/de-doping properties of the PANI materials and the π-π conjugation effect between the MWCNTs and PANI (Figure 6). The FT-IR spectra before and after NH_3_ adsorption were studied. The results show that the peak at 1405 cm^−1^ enhances after ammonia adsorption, which is attributed to the expansion and contraction vibration of the quinone ring. Moreover, the peak at 1238 cm^−1^ weakens, which is attributed to C-NH^+^ stretching (Figure S7) [44]. This is because PANI materials synthesized under acidic conditions are in the emeraldine salt (ES) state and have abundant active sites. When the PANI-MWCNTs/PDMS film sensor is exposed to NH_3_, -NH on PANI combines with NH_3_, which changes PANI from the ES state to the emeraldine base (EB) state and leads to an increase in resistance [14]. When the sensor is placed in the air, NH_3_ detaches from the surface of PANI, and the resistance of the sensor is restored to its original state. The MWCNTs in the film can be used as an efficient sensing channel to enhance the charge transport rate. The existence of the π-π conjugation effect between the MWCNTs and PANI accelerates electron migration and, thus, improves the sensing performance [6,45,46]. In addition, the MWCNTs exposed in the composite film also interact with NH_3_. The MWCNTs embody the characteristics of a p-type semiconductor. When exposed to NH_3_, the hole density of the MWCNTs decreases, and the resistance increases [47,48]. Based on the above points, the gas sensing performance of the film sensor is significantly improved.

After the addition of NH_3_ in a high humidity environment, the initial resistance of the sensor decreases and then increases with the relative humidity. The I-V curves of the PM2 film also prove this phenomenon in different RH environments (Figure S8). The conductivities of the film decrease as the humidity increases. This is because the PANI material swells after absorbing water molecules [48]. The swelling effect of the polymer may cause the PANI chain to twist and increase its disorder, resulting in a restricted charge carrier movement, which increases the resistance of the sensor. The response value of the sensor to NH_3_ increases with an increase in the humidity of the environment. The reason for this result is that water vapor is a weakly reducing gas, and it can promote the adsorption of NH_3_ on PANI materials. The water molecule generates OH^-^ during the adsorption process, which can trap H^+^ on the PANI chain in a high humidity environment to improve the response of the sensor [29,48,49,50].

### 3.4. Applications of the PANI-MWCNTs/PDMS Film Sensor

**Figure 7:**
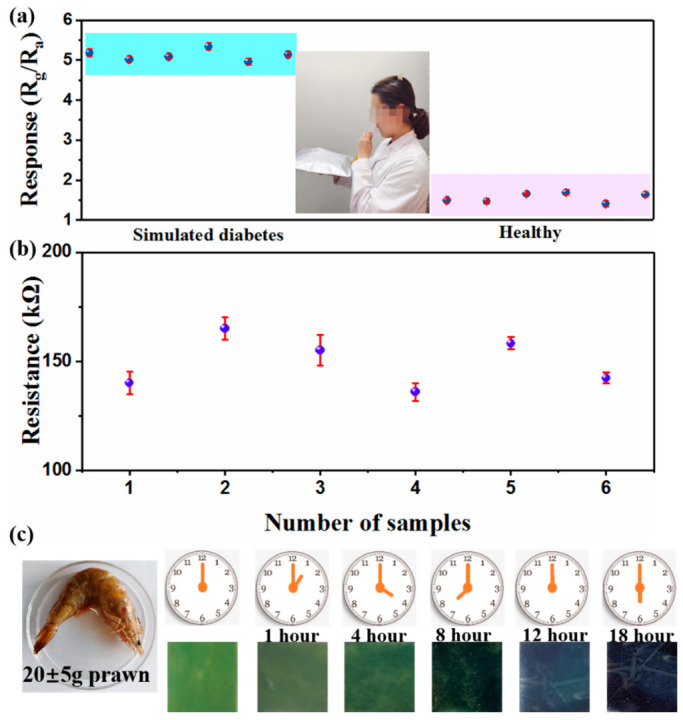
The response of PM2 sensors to exhaled breath of healthy patients and simulated patients with kidney disease (**a**) and the initial resistance of sensor without NH_3_ (**b**); changes in the colorimetric sensors at different shrimp placement times (**c**).

To investigate the potential of the PANI-MWCNTs/PDMS film-based sensor for monitoring expiratory markers, related experiments were performed. The exhaled breath of six healthy people was collected and injected into glass bottles, and one of the glass bottles was injected with 1 ppm NH_3_. Then, the sensor was set into two glass bottles to measure its response. Figure 7 shows the response to NH_3_ and the initial resistance of the sensor. The resistance value of the sensor was between 140 and 115 kΩ, its response value was about 5.0, and the relative standard deviation (RSD) was 9.5%. Without 1 ppm NH_3_, the response value of the sensor to the exhaled breath was about 1.4~1.6. The results indicate that the PANI-MWCNTs/PDMS film-based sensor has a good stability in practical NH_3_ detection, which further proves that PANI-MWCNTs/PDMS sensors have certain advantages and potentials in the diagnosis of nephropathy. However, in practical applications, it is necessary to consider the impact of humidity on the sensor, for which a drying device can be added in front of the test chamber to achieve the effect of water removal [51]. In the process of spoilage, prawn releases a lot of NH_3_ [52,53]. Figure 7b exhibits the color change of the film sensor in the atmosphere of prawn at 28 ± 2 °C to evaluate its freshness. Here, green represents fresh, blue represents petty spoilage, and dark blue represents spoilage. As the storage period increased, the color of the PANI film gradually became darker, changing from green to dark green and finally to blue. The reason for this phenomenon is due to the de-doping reaction between NH_3_ and PANI, which changes its structure from the ES state to the EB state. This result indicates a possibility for visual NH_3_ detection.

## 4. Conclusions

In summary, PANI was grown on MWCNTs/PDMS films via chemical oxidation polymerization. The morphology and structure demonstrated the formation of film materials, and a PANI-MWCNTs/PDMS film-based NH_3_ sensor was successfully developed. At room temperature, the response of the film sensor to 40 ppm NH_3_ was 11.8 ± 0.2, with a recovery time of 236 s and a detection limit of 10 ppb. The sensor was applied to identify the breath of healthy patients and simulated patients with kidney disease, and the results show that the sensor has a good ability to identify simulated samples. Meanwhile, a visualized thin-film sensor with a sensitive color change to NH_3_ was developed. Then, the sensitivity mechanism was systematically explained. This work prepared a flexible sensor based on PANI/MWCNTs, which shows good application prospects for the detection of ammonia in the environment and human breath.

## Figures and Tables

**Table 1:** NH_3_ sensing performances of gas sensors based on PANI/MWCNTs materials.

Sensing Material	Substrate	Response	Detection Limit	Ref.
Co_3_O_4_/MoS_2_	Interdigital electrode	1.65 to 5 ppm	1 ppm	[37]
rGO/ZnO	Glass substrate	5.6 to 0.5 ppm	0.5 ppm	[38]
SiNW	Si substrate	75.8% to 100 ppm	100 ppb	[39]
PEA-Bi-Br	Interdigital electrode	1.76 to 30 ppm	0.2 ppm	[40]
PCo	Interdigital electrode	85% to 20 ppm	1 ppm	[41]
PANI/MWCNTs	Interdigitated array electrode	15.5% to 2 ppm	2 ppm	[18]
CNTs/PANI	Interdigital electrode	1.52 to 40 ppm	4 ppm	[32]
PDA-CNT-PANI	Ceramic tube	1.41 to 10 ppm	38 ppb	[42]
MWCNT-PANI/PVDF	Film	1.33 to 1 ppm	1 ppm	[43]
MWCNTs/PANI/PET-NH_2_	Film	2.17 to 50 ppm	1.1 ppm	[6]
MWCNTs/PANI	Fabric	≈2 to 100 ppm	200 ppb	[44]
CNTs/PANI/PET	Film	about 25 to 50 ppm	200 ppb	[24]
PANI/MWCNTs-PDMS	Film	11.8 to 40 ppm	10 ppb	This work

## Data Availability

The authors confirm that the data supporting the findings of this study are available within the article [and/or its supplementary materials].

## Supplementary Materials

The following supporting information can be downloaded at https://www.mdpi.com/article/10.3390/nano13071158/s1, Figure S1: The schematic of the sensor test system; Figure S2: Picture of MWCNTs/PDMS film; Figure S3: SEM images of PM1 (a,b) and PM3 (c,d) films; Figure S4: Raman spectrograms of PDMS (a) and MWCNTs/PDMS (b) films; Figure S5: The response-recovery curve of PM2 sensor to 10 ppb NH_3_; Figure S6: The response value of PM2 sensor to 10-80% RH; Figure S7: FT-IR spectrum before and after adsorption of NH_3_; Figure S8: I–V curve of the PM2 films at 10–80% RH.
