# Supplementary material for: Flexible Room-Temperature Ammonia Gas Sensors Based on PANI-MWCNTs/PDMS Film for Breathing Analysis and Food Safety
Source: Nanomaterials (Basel). 2023 Mar 24;13(7):1158. doi: 10.3390/nano13071158 (PMC10097228; doi:10.3390/nano13071158)
Supplement: Supplementary file 1 [file nanomaterials-13-01158-s001.zip › nanomaterials-2300347-supplementary.pdf]

Flexible Room-Temperature Ammonia Gas Sensors Based on PANI-MWCNTs/PDMS Film for Breathing Analysis and Food Safety

Chonghui Zhu, Tingting Zhou, Hong Xia * and Tong Zhang *

State Key Laboratory of Integrated Optoelectronics, College of Electronic Science and Engineering, Jilin University, Changchun 130012, China
* Correspondence: hxia@jlu.edu.cn (H.X.); zhangtong@jlu.edu.cn (Tong Zhang)

FIGURE CAPTIONS

Figure S1 The schematic of the sensor test system

Figure S2 Picture of MWCNTs/PDMS film

Figure S3 SEM images of PM1 (a, b) and PM3 (c, d) films

Figure S4 Raman spectrograms of PDMS (a) and MWCNTs/PDMS (b) films

Figure S5 The response-recovery curve of PM2 sensor to 10 ppb NH_3

Figure S6 The response value of PM2 sensor to 10-80% RH

Figure S7 FT-IR spectrum before and after adsorption of NH_3

Figure S8 I-V curve of the PM2 films at 10-80% RH

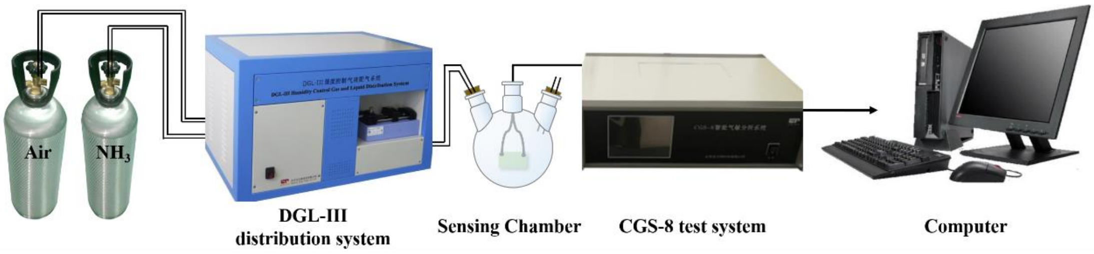

Figure S1 The schematic of the sensor test system

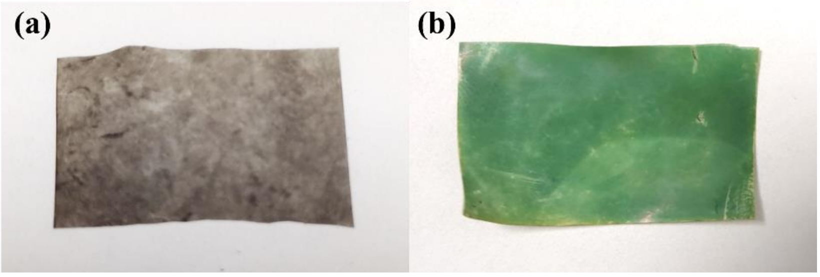

Figure S2 Picture of MWCNTs/PDMS film (a) and PANI/PDMS (b)

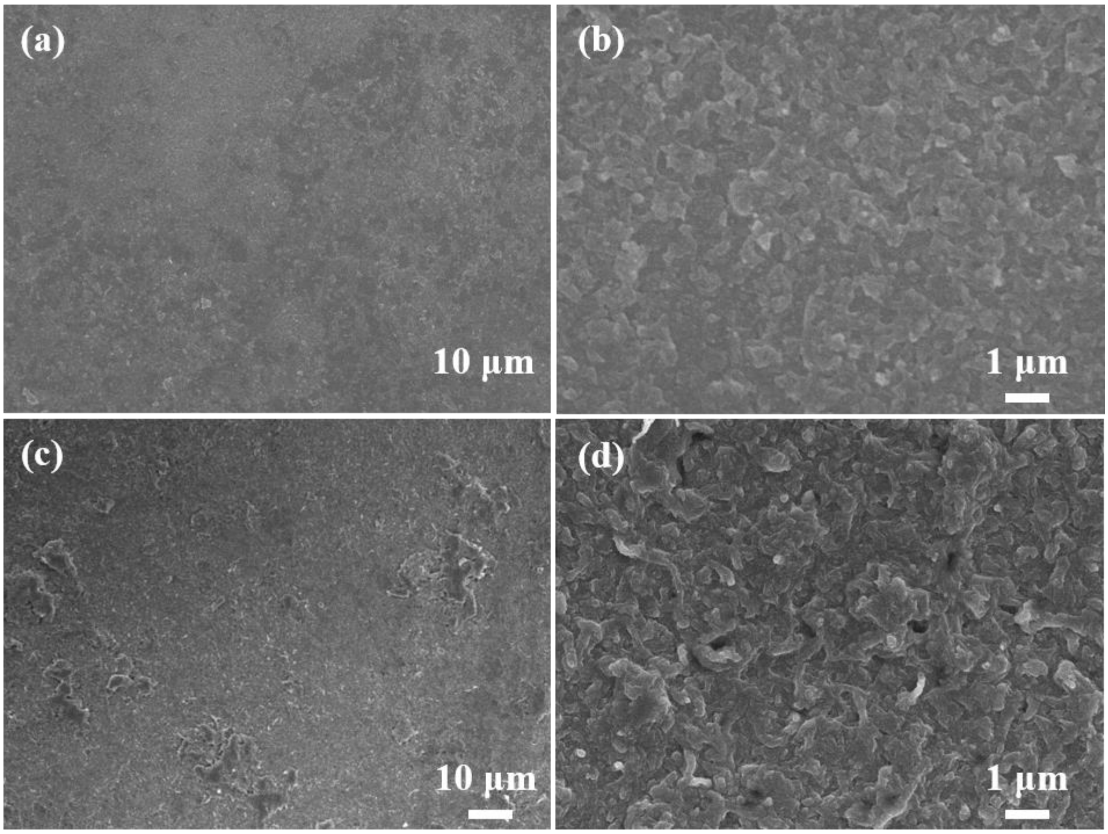

Figure S3 SEM images of PM1 (a, b) and PM3 (c, d) films

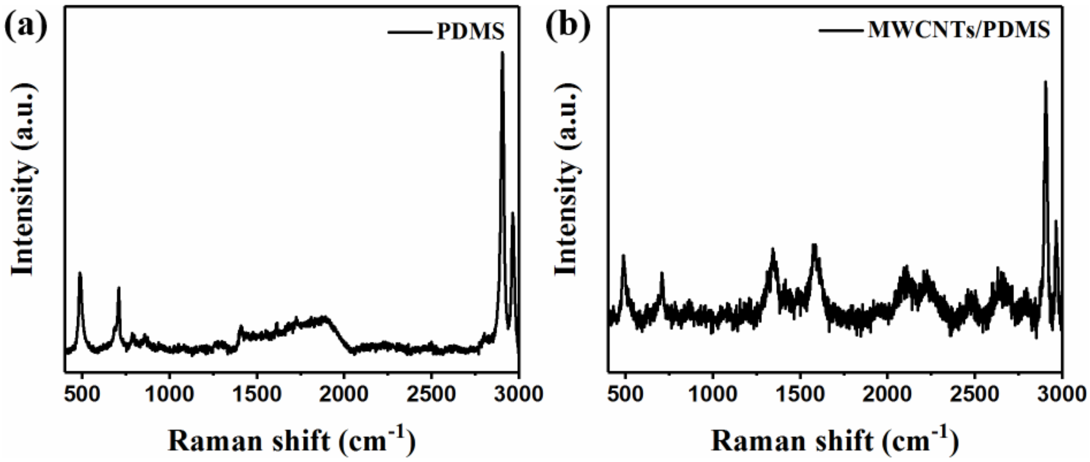

Figure S4 Raman spectrograms of PDMS (a) and MWCNTs/PDMS (b) films

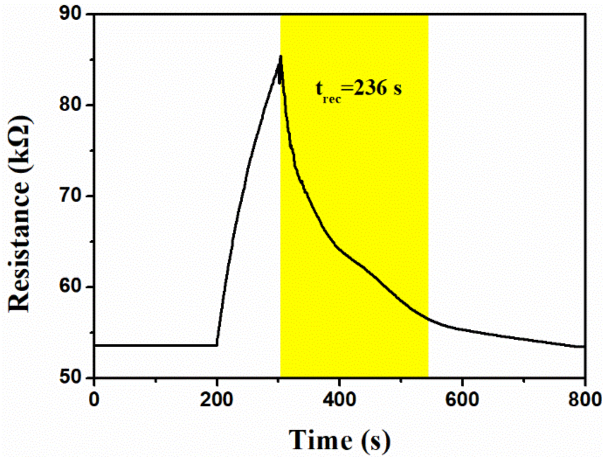

Figure S5 The response-recovery curve of PM2 sensor to 10 ppb NH_3

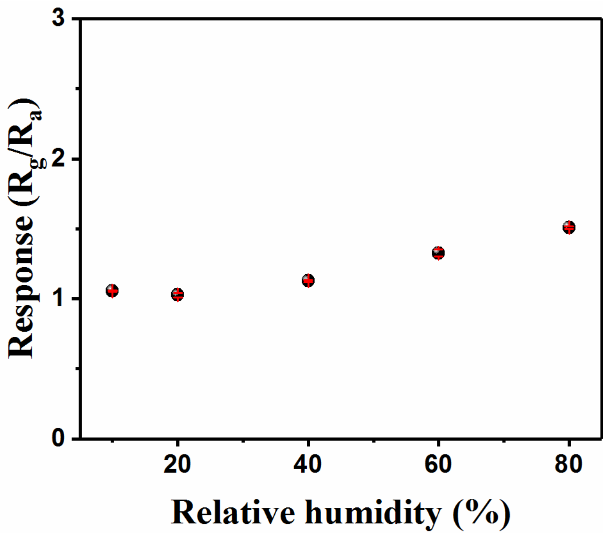

Figure S6 The response value of PM2 sensor to 10-80% RH

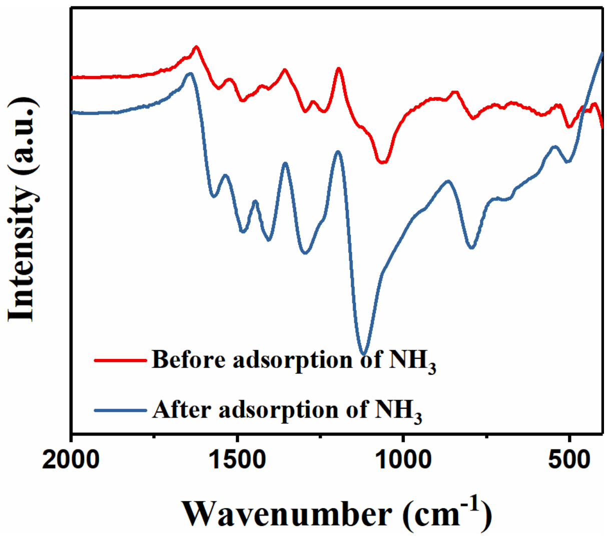

Figure S7 FT-IR spectrum before and after adsorption of NH_3

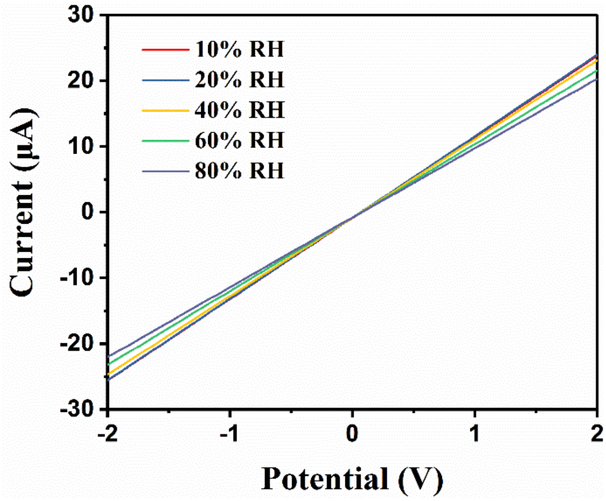

Figure S8 I-V curve of the PM2 films at 10-80% RH
